# The challenge of prognostic markers in acute pancreatitis: internist’s point of view

**DOI:** 10.1186/s43141-021-00178-3

**Published:** 2021-05-25

**Authors:** Ombretta Para, Lorenzo Caruso, Maria Teresa Savo, Elisa Antonielli, Eleonora Blasi, Fabio Capello, Tiziana Ciarambino, Lorenzo Corbo, Armando Curto, Margherita Giampieri, Lucia Maddaluni, Giacomo Zaccagnini, Carlo Nozzoli

**Affiliations:** 1 Medicina per la complessità assistenziale 1 AOU Careggi, Largo Piero Palagi, 1, 50139 Florence, Italy; 2 Società Italiana di Salute Digitale Telemedicina, via Teodoro Valfrè, 00165 Rome, Italy; 3 International Study Center of Society of Telemedicine and Digital Health, 40100 Bologna, Italy; 4 Presidio Ospedaliero Marcianise UOC Medicina Interna, Viale Sossietta Scialla, 81025 Marcianise, ASL, Caserta, Italy; 5 Clinic Gastroenterology, AOU Careggi, Largo Piero Palagi, 1, 50139 Florence, Italy

**Keywords:** Acute pancreatitis, Prognostic factors, Score, Internal wards, Outcome

## Abstract

Acute pancreatitis, the most frequent hospitalization reason in internal medicine ward among gastrointestinal diseases, is burdened by high mortality rate. The disease manifests mainly in a mild form, but about 20-30% patients have a severe progress that requires intensive care. Patients presenting with acute pancreatitis should be clinically evaluated for organ failure signs and symptoms. Stratifying patients in the first days from symptoms onset is essential to determine therapy and care setting. The aim of our study is to evaluate prognostic factors for acute pancreatitis patients, hospitalized in internal medicine wards, and moreover, understanding the role of various prognostic scores validated in intensive care setting in predicting in-hospital mortality and/or admission to intensive care unit. We conducted a retrospective study enrolling all patients with diagnosis of acute pancreatitis admitted took an internal medicine ward between January 2013 and May 2019. Adverse outcome was considered in-hospital mortality and/or admission to intensive care unit. In total, 146 patients (137 with positive outcome and 9 with adverse outcome) were enrolled. The median age was (67.89 ± 16.44), with a slight prevalence of male (55.1%) compared to female (44.9%). C protein reactive (p = 0.02), creatinine (p = 0.01), sodium (p = 0.05), and troponin I (p = 0.013) after 48 h were significantly increased in patients with adverse outcome. In our study, progression in SOFA score independently increases the probability of adverse outcome in patients hospitalized with acute pancreatitis. SOFA score > 5 is highly predictive of in-hospital mortality (O.R. 32.00; C.I. 6.73-152.5; p = 0.001) compared to other scores. The use of an easy tool, validated in intensive care setting such as SOFA score, might help to better stratify the risk of in-hospital mortality and/or clinical worsening in patients hospitalized with acute pancreatitis in internal medicine ward.

## Introduction

The number of hospitalizations for acute pancreatitis (AP) has doubled over the two last decades [1, 2]. AP is the leading cause of hospitalization in the USA in internal medicine wards among gastrointestinal diseases and is burdened by a high mortality rate [3, 4]. In most cases, the disease manifests in a mild form, but about 20-30% of patients have a severe evolution associated with a single or multi-organ failure that requires intensive care. An early assessment of severity in AP is crucial, the initial 12 to 48 h of hospitalization are critical for patient management, as this period is considered a window of opportunity for defining interventions to prevent pancreatic necrosis and organ failure [5, 6]. The challenge is indeed to assess AP severity during its early stages in order to prevent complications. Extensive researches have already focused on risk and prognostic factors. Nevertheless, none of the current clinical scoring systems or biochemical markers plays a definitive role, has widespread applicable value or is consistently accurate [7, 8]. The aim of our study is to assess the prognostic factors of patients hospitalized for AP in a specific ward, internal medicine. We also evaluate the efficacy of different prognostic scores, validated in setting different from internal medicine in predicting mortality among patients with AP diagnosis.

## Materials and methods

### Design of study

We conducted a retrospective study on patients admitted between January 2013 and May 2019 to an internal medicine ward in AOU Careggi, in Florence, Italy. The patients were enrolled through a computerized electronic medical record (Archimed® medical software version 6.20 by B. Dannaoui, Florence, Italy).

#### Inclusion criteria

We enrolled patients, transferred from emergency department to internal ward, whose clinic and radiological findings did not suggest the need of high intensity care at first assessment. The patients enrolled were both males and females, aged above 18 years old. We enrolled all the patients with diagnosis of AP during the recovery or at the entrance in ward. According to the revised Atlanta classification, the diagnosis of AP requires at least 2 of the following features [9]:

(a) Characteristic abdominal pain

(b) Biochemical evidence of pancreatitis (amylase or lipase elevated > 3 times the upper limit of normal)

(c) Radiographic evidence of pancreatitis on cross-sectional imaging

#### Exclusion criteria

Patients who did not satisfy Atlanta classification or needed high intensity care at first assessment were excluded.

All the procedures performed in this study were in accordance with the ethical standards of the institutional or national research committee and with the 1964 Helsinki Declaration and its later amendments or comparable ethical standards. This is a retrospective study that include anonymized patients extracted from hospital database; data are presented aggregated and anonymously. No informed consent was taken.

We also categorized AP presentation as mild, moderately severe, or severe, based on Atlanta classification [9].

Patients were divided in two groups: those with positive outcome (discharged) and those with adverse outcomes (died or transferred to an intensive care unit).

For each enrolled patient were collected: personal data, admission data; comorbidity (gallstones disease, hypertension, heart failure, chronic obstructive pulmonary disease, chronic kidney disease, diabetes mellitus, previous pathology cerebrovascular disease, dementia, presence of solid or hematological neoplasia, alcoholism and smoking habit, body mass index), laboratory analysis (creatinine, sodium, potassium, calcium, brain natriuretic peptide (BNP), troponin I, protein C reactive, procalcitonin, hematocrit, albumin) on the first admission day and after 48 h, arterial blood gas (ABG) parameters at admission and after 48 h, antibiotic and fluid therapy during hospitalization, home therapy. We also computed for each patient the following score, validated for AP in different setting from internal ward: RANSON, APACHE II, MEWS, SOFA score, Q-SOFA, BISAP, CCI, HAPS, ECOG/PS score, BALTHAZAR.

### Statistical analysis

Continuous variables were expressed through means and standard deviation, while dichotomous variables were expressed like number and percentage of patients. The Student’s t test was used to compare continuous variables, whereas the chi-squared test was used for the comparison of non-continuous variables. We used Shapiro-Wilk test to verify continuous variables normality. We performed univariate analysis to examine the contribution of the variables in predicting the chosen outcome. The results were considered statistically significant for values of p < 0.05 and 95% of the confidence interval (C.I.). The receiver operating characteristic (ROC) analysis was used to obtain the most accurate cut-off of some single continuous variables. We performed logistic regression multivariate analysis (using a stepwise forward regression model, with an entry probability for each variable set at 0.05) to assess the independent contribution of the variables in predicting the chosen outcome. Statistical analysis was conducted with SPSS statistical software version 20.0 (SPSS Chicago, IL).

## Results

Between 1 January 2013 and 20 May 2019, 12,499 patients were admitted to Internal Medicine AOU Careggi department. Of these, 146 patients with AP (137 with positive outcome and 9 with adverse outcome) were enrolled. The median age was 67.89 ± 16.44, with a slight prevalence of male (55.14%) compared to female (44.9%). Stratifying the patients according to classification of Atlanta, we find 122 mild pancreatitis, 8 moderately severe, 16 severe. The main cause of AP resulted the gallstones disease (59.18%), followed by alcoholism (7.48%), and post ERCP-pancreatitis (7.56%). Patients with severe AP were significantly older than patients with mild-moderate pancreatitis (p = 0.025) (Table 1).

For each group, we examined the main comorbidities associated to AP. Patients with chronic kidney disease had acute kidney injury in 56% of cases. Characteristically, the average creatinine serum was higher in patients with severe AP (p = 0.014) (Table 2).

Regarding the laboratory analysis, there were no significative differences between two groups at the admission in the hospital. As it is seen in Table 3 C-reactive protein (CPR) (p = 0.02), creatinine (p = 0.01), sodium (p = 0.05), and troponin I (p = 0.013) after 48 h from the admission were significantly increased in patients with adverse outcome.

**Table 1:** Demographic characteristic in the two groups

Demographic characteristic	Positive outcome	Adverse outcome	P
Age	67.49 ± 16.28	71.67 ± 18.75	0.46
Male	77 (52.7%)	4 (2.7%)	/
Female	60 (41.1%)	5 (3.4%)	/
B.M.I.^1^	26.95 ± 5.58	26.78 ± 4.82	0.939

^1^*B.M.I* body mass index

**Table 2:** Comorbidities in the two groups

Comorbidity	Positive outcome	Adverse outcome	Total
Chronic renal failure	13 (9.4%)	3 (33.33%)	16 (10.88%)
Dementia	15 (10.95%)	3 (33.33%)	18 (12.92%)
Gallstone disease	81 (59.6%)	5 (55.6%)	86 (59.18%)
Hematological neoplasia	5 (3.7%)	1 (11.1%)	6 (4.08%)
Solid neoplasia	31 (22.8%)	2 (22.2%)	33 (22.45%)
Previous TIA^1^ or stroke	13 (9.6%)	13 (9.6%)	16 (10.88%)
COPD^2^	7 (5.1%)	1 (12.5%)	8 (5.5%)
Heart failure	17 (12.5%)	2 (22.5%)	19 (13.60%)
Hypertension	80 (58.8%)	5 (55.6%)	85 (58.50%)
Diabetes	27 (19.9%)	2 (22.2%)	29 (19.73%)

^1^*TIA* transient ischemic attack,

^2^*COPD* chronic obstructive pulmonary disease

**Table 3:** Blood samples after 48 h

Blood samples after 48 h	Positive outcome	Adverse outcome	P
C reactive protein (mg/dL)	87.03 ± 83.4	240 ± 61.24	**0.02**
Procalcitonin (ng/mL)	10.37 ± 46.92	4.53 ± 7.61	0.747
Heart troponin I (ng/mL)	0.053 ± 0.965	0.32 ± 0.64	**0.013**
Hematocrit (%)	36.47 ± 4.66	34.26 ± 9.74	0.232
Creatinine (mg/dL)	1.08 ± 0.897	1.96 ± 1.755	**0.01**
NT-pro BNP^1^ (pg/ml)	3826.39 ± 10,497.48	11,174 ± 17,452	0.471
Potassium (mEq/L)	3.734 ± 0.45	4.022 ± 0.58	0.184
Sodium (mEq/L)	139.41 ± 3.658	143.56 ± 8.22	**0.05**
Calcium (mg/dL)	8.35 ± 0.73	8.08 ± 0.64	0.386
Albumin (g/L)	33.33 ± 8.13	25.4 ± 9.96	0.301

^1^N-terminal prohormone of brain natriuretic peptide

Regarding ABG, after 48 h from admission, more acid pH was associated with a negative outcome (p = 0.027) (Table 4).

**Table 4:** pH and lactate at admission and after 48 h

	Positive outcome	Adverse outcome	P
**A.B.G. at admission**			
pH	7.44 ± 0.08	7.36 ± 0.075	**0.05**
Lactate	7.61 ± 19.37	7.98 ± 8.94	0.939
**A.B.G. after 48 h**			
pH	7.43 ± 0.047	7.37 ± 0.104	**0.027**
Lactate	5.64 ± 4.20	11.7 ± 11.17	**0.05**

**Fig. 1:**
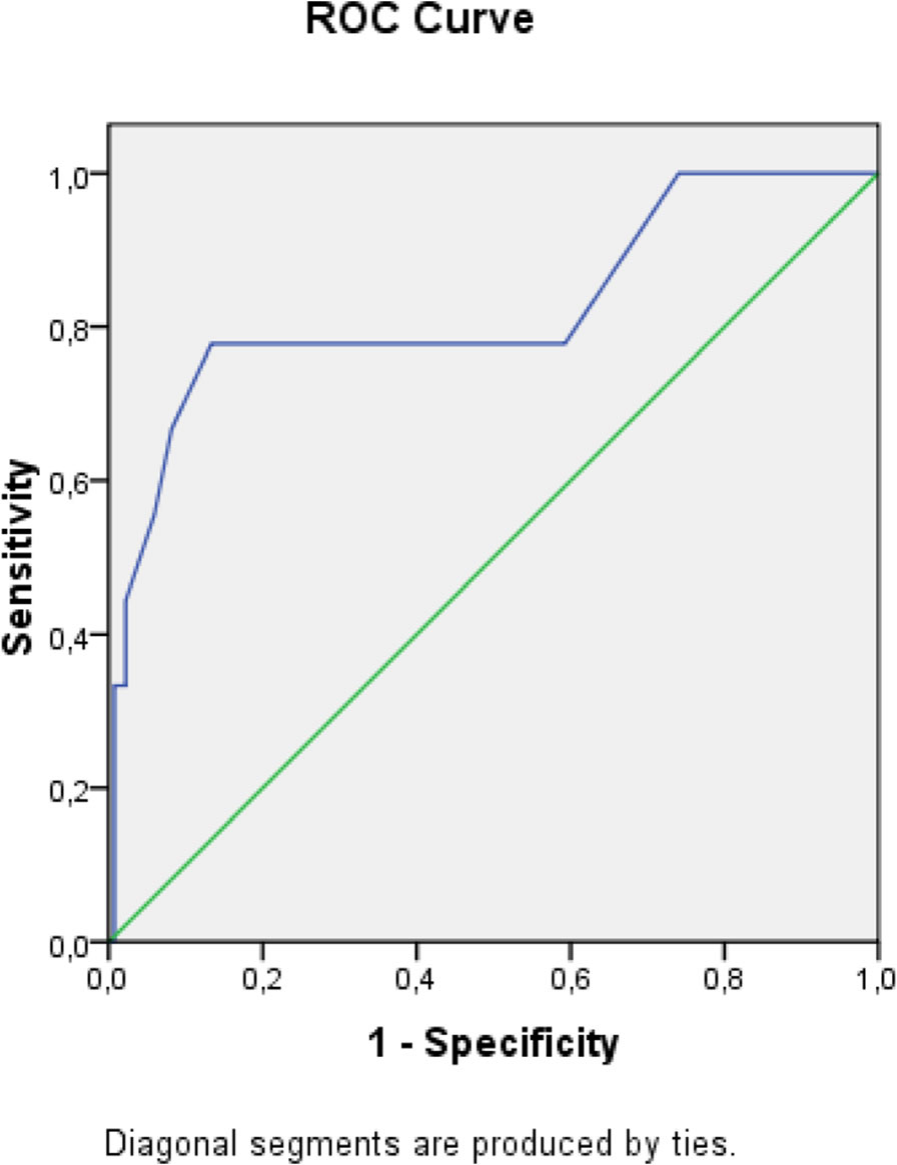
ROC Curve for SOFA score

**Fig. 2:**
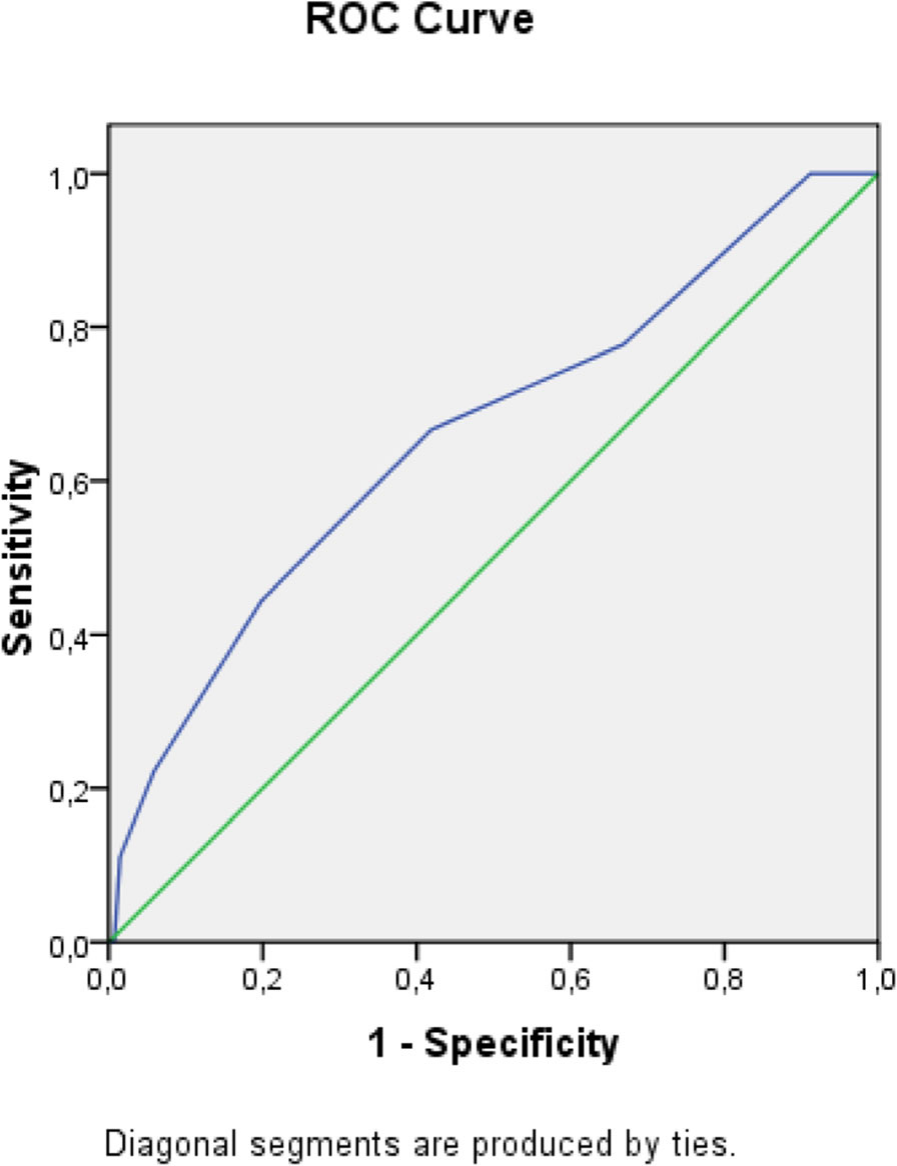
ROC curve for Ranson score

**Fig. 3:**
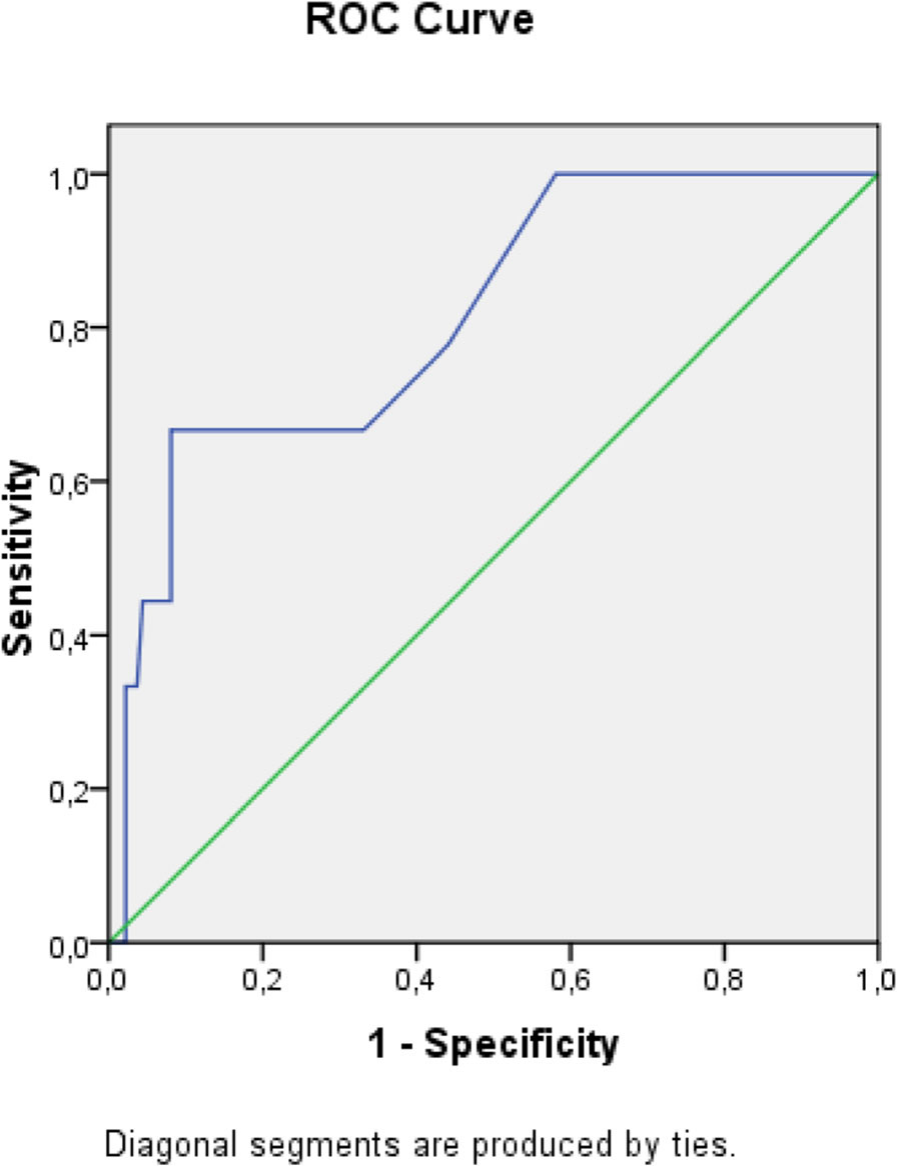
ROC curve for APACHE II score

**Fig. 4:**
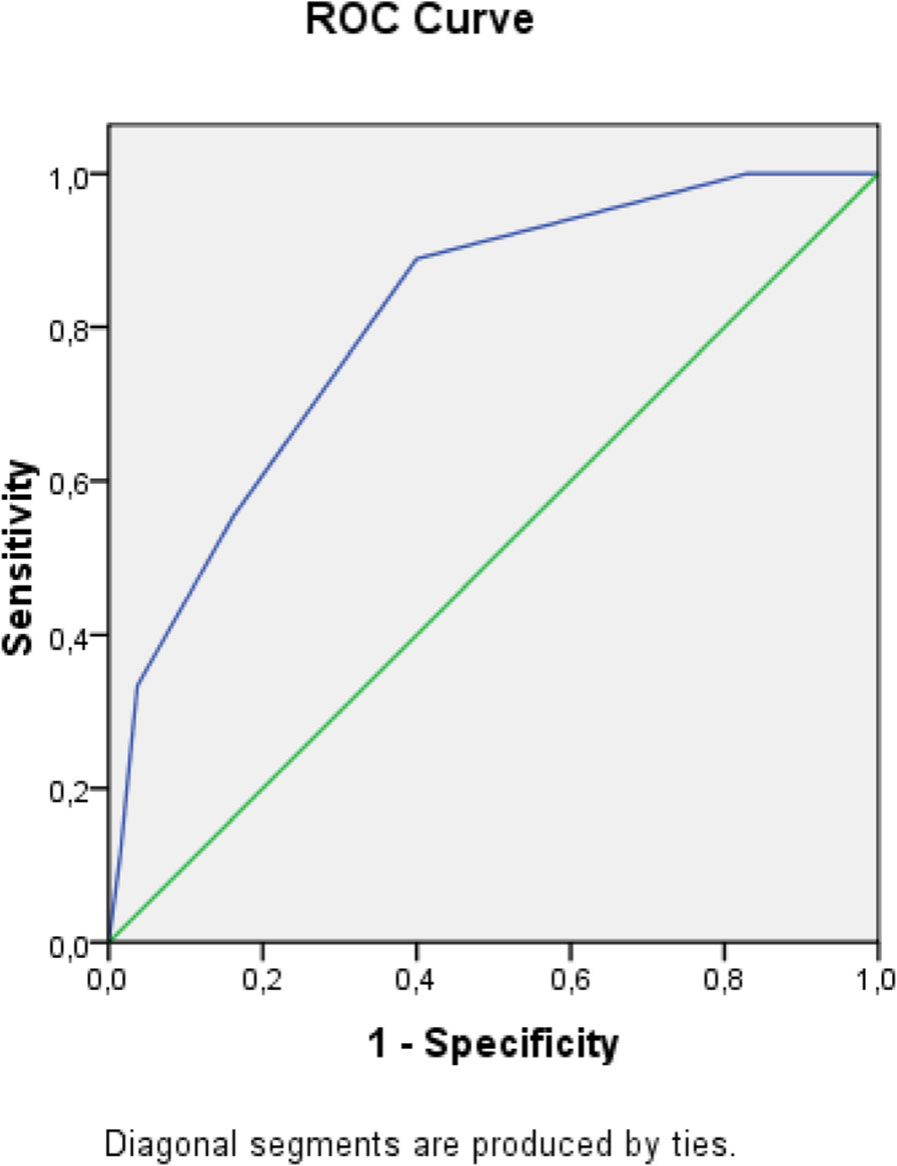
ROC curve for BISAP score

**Fig. 5:**
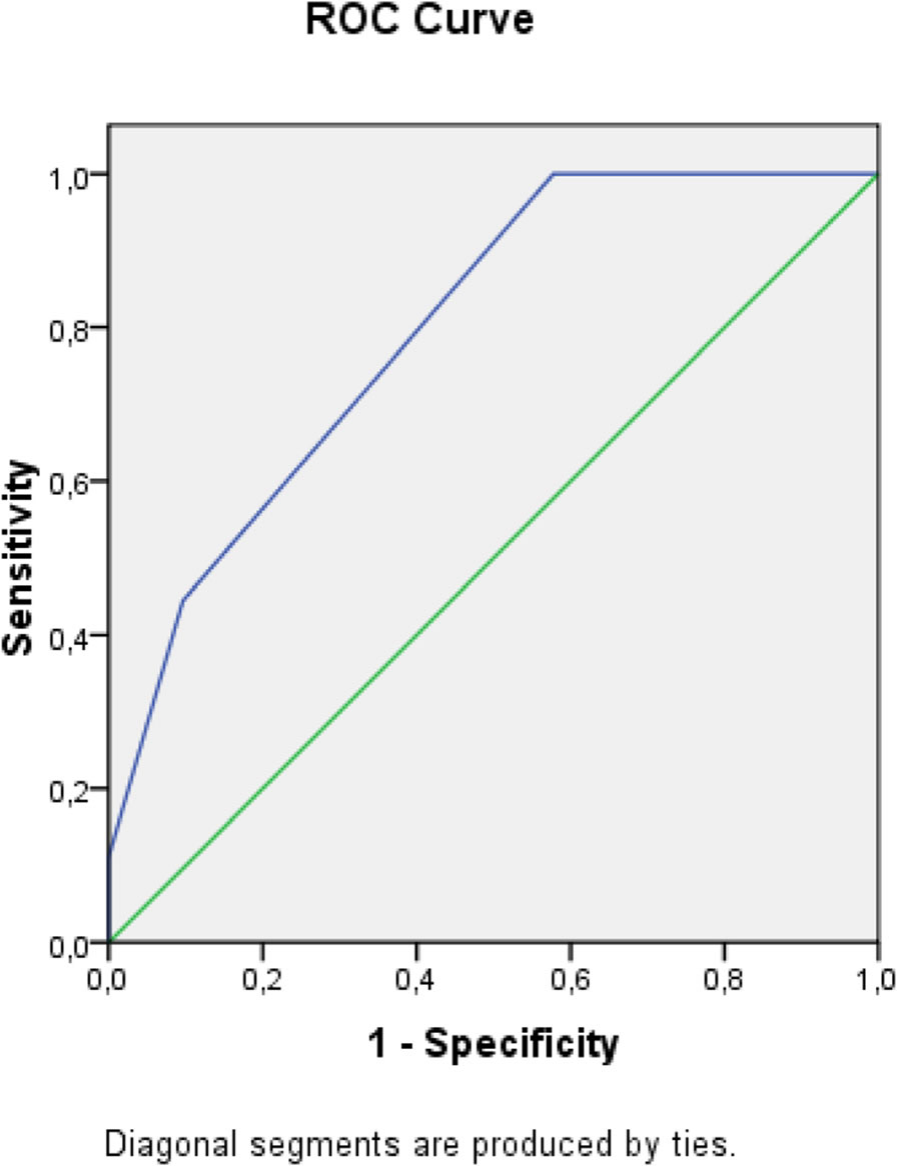
ROC curve for HARMLESS score

**Fig. 6:**
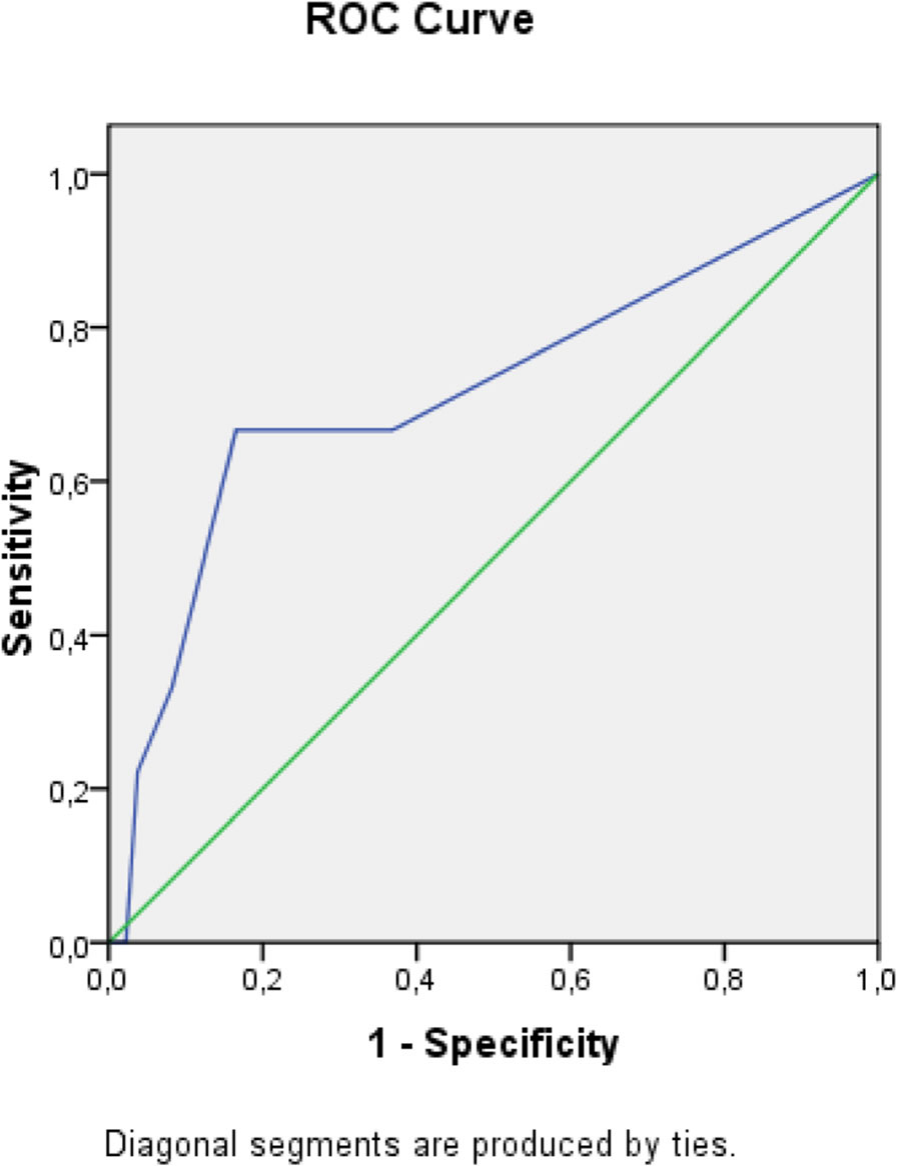
ROC curve for MEWS score

**Fig. 7:**
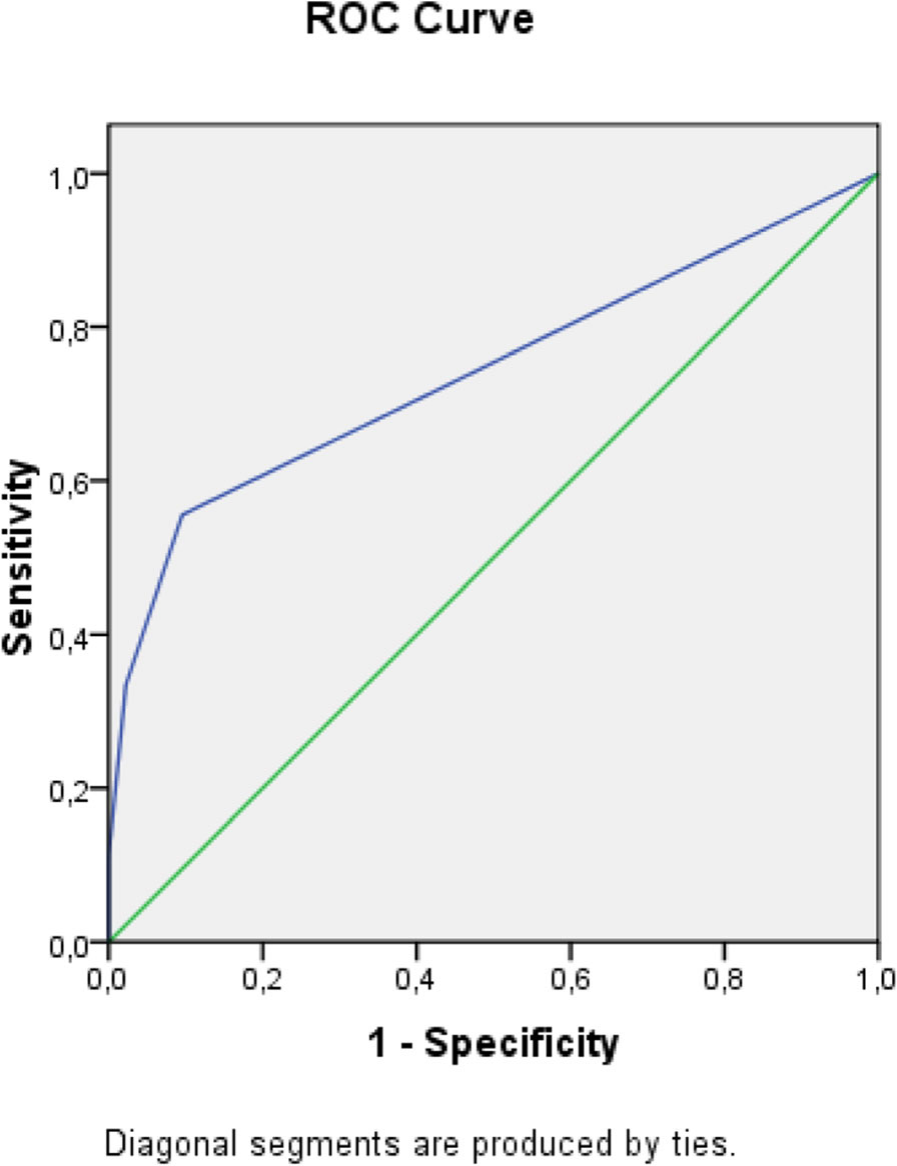
ROC curve for Q-SOFA

The receiver operating characteristic (ROC) analysis was used to obtain the most accurate cut-off for the score significantly different between two groups: values above the cut-off were associated to a poor prognosis (Figs. 1, 2, 3, 4, 5, 6, and 7) (Table 5).

**Table 5:** Cut off determined for each score

Score	Cut-off	O.R.	C.I. > 95%	P
**APACHE II**	> 13	9.531	2.32-39.21	**0.001**
**MEWS**	> 2	4.808	1.07-21.52	**0.026**
**SOFA score**	**> 5**	**32.000**	6.73-152.5	**0.001**
**Q-SOFA**	**> 1**	16.625	3.02-91.54	**0.001**
**BISAP**	**> 3**	4.218	1.05-16.96	**0.03**
**HARMS**	**> 1**	4.429	0.996-19.69	**0.035**
**BALTHAZAR**	**> 3**	6.225	1.25-31.34	**0.012**

SOFA score > 5 was the most accurate to predict adverse outcome (O.R. 32.000, **p = 0.001)**; q-SOFA score > 1 also showed a good correlation with adverse outcome (O.R. 16.625, **p = 0.001**).

In patients with adverse outcome, more aggressive fluid therapy (2777.8 ml ± 1545.545) was administered compared to patients with a positive outcome (1772.96 ± 763 ml) (**p = 0.001**).

We also conducted a multi-variate analysis whose results are not reported because they were not statistically significative.

## Discussion

In our case study, the mortality rate resulted 2.77%, similar to data reported in literature (the mortality rate changes from 2.5% for mild AP to 30% for moderate-severe form 30%) [1, 10, 11].

According to classification of Atlanta, we stratify 122 mild pancreatitis, 8 moderately severe, and 16 severe. Severe AP was observed in older patients as elderly age is an important prognostic factor [12, 13]. Indeed, older patients have more frequently organ failure during AP, probably due to intestinal bacterial translocation [14]. The increase of intestinal bacterial translocation is correlated to a pro-inflammatory status [15–17] called *inflammaging*, which is the reason of a lack immune system response to antigens [18]. Cellular aging in older patients is also an important cause of poor immune response [15, 19].

The first aim of our study was to evaluate blood samples analytes to find prognostic factors: increase of C-reactive protein, creatinine, sodium and troponin I, and reduction of pH after 48 h from the onset of symptoms result as prognostic factors in patients with adverse outcome.

Troponin I is an important heart damage marker but its diagnostic capacity is more quantitative than qualitative: according to recent European Society of Cardiology guidelines, increment of troponin should be at least three times higher than 99 percentile of reference to be diagnostic of heart injury [16]. The increase of troponin levels, in patients with normal electrocardiogram and in absence of chronic renal failure, is compatible with acute pancreatitis in the first 72 h from the onset of symptoms [17]. We speculate that the higher value of troponin should be due to asymptomatic rhabdomyolis which is one of the least known and recognized complications of AP [20].

Patients with poor outcome had an elevated value of C-reactive protein. During the first phase of acute pancreatitis, cytokine pro-inflammation, as TNF (tumor necrosis factor) α, interleukins (IL-1, IL-2, IL-18, IL-6) [21] and chemokine, oxygen radical oxidant are released [22]. In mild acute pancreatitis, immune system of patients limits inflammation in the pancreatic area. In severe form of AP, massive damage and inflammation determine systemic inflammatory response syndrome (SIRS) [23]. The systemic inflammation causes a release of cytokine in blood circulation [24, 25]. In the liver, IL-6 is a very powerful indicator of C-reactive proteins and procalcitonin [26].

The increase of creatinine serum in patient with AP has been largely studied [27]. Acute kidney injury (AKI) can be a systemic complication of AP; pancreatic amylase can damage renal microcirculation, causing both hypoxic and toxic damage due to pancreatic phospholipase A2 action on proximal tubule. All these factors contribute to reduce renal perfusion, to damage tubules, and to increase creatinine serum concentration [28]. The AKI incidence in patients with AP is between 1 and 15% and it is an important cause of death with percentage between 0 and 30% [29].

In patients with adverse outcome, ABG after 48 h from admission reported an average pH more acidic than patients with positive outcome. We speculate that extracellular acidosis determines release of cytokines as interleukin 1β from the immune cells [30, 31] causing necrosis and further inflammation in patients with AP [32].

Secondary aim of our study was the assessment of different prognostic scores (in Table 6, we reported a brief introduction to the main scores) in predicting mortality and need of high intensity care among patients with AP admitted in internal ward. For each patient, we calculated the main score validated in setting different from internal medicine, as emergency department, or high intensity unit care. Although SOFA score, quick-SOFA, and APACHE II were validated in different setting, their application in internal ward demonstrated the most accurate relationship with patients’ outcome. Also, Tee and Fang [33] compared RANSON, APACHE II, and SOFA score demonstrating similar sensibility and specificity in predicting mortality in severe acute pancreatitis after 48 h from the admission.

**Table 6:** A brief introduction to scores

Score	Setting	Specific^*^	Usual cut-off^**#**^	Cut-off for AP in our study
**APACHE II**	Intensive unit care	Designed for AP, then used in intensive setting for different disease	> 8	**> 13**
**MEWS**	Clinic score, at patient’s bed, in emergency	Used everywhere for its ease.	> 3	**> 2**
**SOFA score**	Intensive unit care	Sepsis and the multi-organ failure	Each result correlates to a higher risk of mortality	**> 5**
**Q-SOFA**	Intensive unit care	As SOFA score but simple to assess	> 2	**> 1**
**BISAP**	At patient’s bed	Designed for AP, in the first hours from hospitalization	As SOFA, not a really cut-off but a higher risk of mortality	**> 3**
**BALTHAZAR**	Radiological score obtained with computed tomography	Specific for AP, correlates to complication, local, and systemic	> 2	**> 3**

*Designed or not for AP

^#^A superior value is associated to a poor prognosis

SOFA score > 5 shows the best correlation to a poor outcome. SOFA score is a useful tool to evaluate patients admitted in high intensity unit care; however, its use is reduced in low-care setting such as internal wards; for this reason, SEPSIS-3 [34] guidelines suggest to use rather q-SOFA, more easily and quickly applicable to assess septic patient prognosis. The application of q-SOFA demonstrated more elevated value in patients with poor outcome recovered in internal wards. The main limit of q-Sofa is underestimating the gravity of illness which can determine undertreatment [35].

In our study, RANSON score was not associated with adverse outcome. Also, recent literature support a better prognostic capacity for APACHE II e BISAP [36, 37].

Patients with negative outcome received more aggressive fluids therapy compared to patients with a positive outcome. Although fluid therapy is one of the hinges in the AP management to prevent hypovolemia and hypoperfusion of tissues, data on the amount of fluid needed to prevent necrosis or to improve outcome is contradictory. The amount of liquid must be adjusted to the patient’s age, weight, and pre-existing renal and/or cardiac conditions. More fluid were administered to patients with severe AP because of their hemodynamic impairment.

*American Gastroenterological Association* (*AGA*) guidelines suggest the use of goal direct therapy in patients with AP [38]. Goal-directed therapy is successful in septic patients but in patients with AP there are no reported reduction of mortality rate or risk of multi-organ failure [39]. In addition, respiratory distress and abdominal compartimental syndrome are reported as side effects of a too much aggressive fluid therapy [39, 40]. In two prospective randomized trials, therapy with Ringer lactate had more effectiveness than fluid resuscitation with saline solution in reducing value of C-reactive protein and incidence of SIRS in patients with AP [41, 42]. For the lack of evidence, AGA guidelines do not make recommendation regarding the choice between Ringer’s lactate versus normal saline as the optimal fluid solution for resuscitation. Normal saline solution can increase hypercloremic acidosis, worsening the inflammation and pancreatic necrosis [43]; in the other side, Ringer lactate can reduce inflammation inhibiting macrophages [44] and has a less acid pH (6.5 vs 5.5). Different studies confirmed the role of acid pH into worsening necrosis and pancreatic inflammation [32]. We speculate that Ringer lactate can improve the outcome of patients but the study in support of Ringer lactate has the important limit of poor primary end-point, as organ failure, pancreatic necrosis, and mortality.

It is difficult from data in the literature understanding the relationship between the severity of illness, the quantity of fluids administered and outcome [45, 46]. The difficulties deriven from the retrospective nature of our study which, as the main in literature, show bias about causuality relationship [47].

Our study has some limitations because it is carried out in a single department of a single hospital center and the population examined consists of a limited number of patients. Moreover, patients with severe acute AP from the onset of symptoms were admitted directly to high intensity care unit from emergency department; this implicated reduced sample for patients with negative outcome as only mild and moderate AP at the onset of symptoms were recovered in internal ward. For an internal ward, expanding the poor sample might lead the study for at least 10 years. Further studies in multicenter system should be conducted to assess prognostic factors in patients recovered in internal wards, because of the rapidly evolving AP which makes stratification of patients at the onset of symptoms a real challenge.

## Conclusions

In the setting of internal medicine, acute pancreatitis causes significant mortality rate. Stratifying patients according to prognosis at the onset of symptoms is essential to optimize therapy and establish correct care setting. Our study showed that an increase of C-reactive protein, creatinine, sodium, and troponin I after 48 h from the onset of symptoms are prognostic factors for patients with adverse outcome. Moreover, the main score, validated in different settings from internal medicine demonstrated a good correlationship with our patients’outcome.

## Data Availability

The datasets used and/or analyzed during the current study are available from the corresponding author on reasonable request.

## Abbreviations

A.B.G.: Arterial blood gas
A.P.: Acute pancreatitis
APACHE II: Acute Physiology And Chronic Health Evaluation II
BNP: Brain natriuretic peptide
BISAP: Bedside Index of Severity in Acute Pancreatitis
CCI: Charlson Comorbidity Index
CPR: C-reactive protein
ECOG/PS: Eastern Cooperative Oncology Group/Performance Status
HAPS: Harmless Acute Pancreatitis Score
IL: Interleukin
MEWS: Modified Early Warning Score
Q-SOFA: Quick - Sequential Organ Failure Assessment
SIRS: Systemic Inflammatory Response Syndrome
SOFA: Sequential Organ Failure Assessment
